# Application of Optical and Rheological Techniques in Quality and Storage Assessment of the Newly Developed Colloidal-Suspension Products: Yogurt-Type Bean-Based Beverages

**DOI:** 10.3390/s22218348

**Published:** 2022-10-31

**Authors:** Patrycja Cichońska, Ewa Domian, Małgorzata Ziarno

**Affiliations:** 1 Department of Food Technology and Assessment, Institute of Food Science, Warsaw University of Life Sciences-SGGW (WULS-SGGW), Nowoursynowska 159c St., 02-787 Warsaw, Poland; 2 Department of Food Engineering and Process Management, Institute of Food Science, Warsaw University of Life Sciences-SGGW (WULS-SGGW), Nowoursynowska 159c St., 02-787 Warsaw, Poland

**Keywords:** plant-based beverages, fermentation, storage stability, viscoelastic properties, particle size distribution, oscillatory rheology

## Abstract

The objectives of this study were to compare the properties of the yogurt-type bean-based beverages B and BG produced from the nongerminated and germinated beans, respectively, by high-pressure homogenization (HPH) and fermentation with three starter cultures. Optical techniques were used to evaluate the particle size distribution (PSD), color parameters, and instability during storage, while rheological tests were used to evaluate the shear viscosity, flow behavior, and viscoelastic properties. The BG compared to B, irrespective of the starter culture used, showed a higher mean diameter and Span of PSD (d_4,3_ ≈ 76.8–84.2, Span ≈ 2.24–2.35 for BG vs. d_4,3_ ≈ 38.2–47.0, Span ≈ 1.90–2.00 for B). The BG vs. B showed lower viscosity (0.47 Pa·s for BG vs. 0.81 Pa·s for B at shear rate 75 s^−1^) and slightly lower but satisfactory stability (after 21 days at 6 °C, the Turbiscan Stability Index TSI ≈ 1.3–2.0 for BG vs. TSI ≈ 0.6–0.9 for B). Both B and BG were characterized by light-yellow color and showed the characteristics of a viscoelastic fluid. The HPH and germination mainly affected the properties of the tested plant tissue, which has a direct impact on the properties of the final products.

## 1. Introduction

Foods are complex materials, mostly consisting of mixtures of solids and fluid structural components [1]. When developing a novel food, it is of great importance to determine its properties, which is possible through the use of various types of analytical techniques. Food analysis is an essential aspect of quality control, which enables the production of safe food with defined and reproducible properties [2]. Besides traditional methods for assessing the quality of food, such as chemical, microbiological, and sensory analysis, there are also rheological and optical methods that describe the structural properties of food. 

Most of the optical methods used in food analysis are based on conventional visible and near-infrared spectroscopy. These techniques use measurements of the aggregate amount of light either reflected from or transmitted through a biological or food material, resulting from the combined effect of absorption and scattering of photons by the tissues. The obtained data are converted into quantitative or qualitative parameters describing the observed phenomenon [3]. The optical methods can be used for nondestructive, real-time, and fast monitoring of the samples. They are based on measuring the properties of incident or reflected light in food systems. Various types of analytical devices are used that analyze light scattering, laser diffraction of particles, or monitor the mechanisms that occur in food during storage, including particle destabilization. As a result, optical studies enable the analysis of various food characteristics, including, e.g., stability, color, turbidity, and particle size distribution [4,5]. 

Rheology studies the flow and deformation of matter and is an important tool to characterize fundamental processing properties of food systems such as fluid flow properties, viscosity, and elasticity [6]. It describes a relationship between the stress acting on a given material and the resulting deformation and/or flow that takes place [1,6]. Rheological properties play an important role in food process design. Rheological data are required for calculation in any process involving fluid flow, including pump sizing, extraction, filtration, extrusion, and purification. These data enable an analysis of flow conditions in many food processes (e.g., in pasteurization, evaporation, and drying) [5,7].

Plant-based diets have been gaining popularity in the food market in recent years. As reported in previous studies, consumers are increasingly avoiding animal products due to the following reasons: allergies to the proteins present in cow’s milk, intolerance to lactose, environmental or animal welfare concerns, desire to follow a vegan lifestyle, and willingness to diversify their diets [8,9,10,11]. Among dairy alternatives, the most popular ones are milk substitutes, which are usually referred to as plant-based beverages [12]. These products are colloidal systems that are formed by large, dispersed particles such as fat globules, solid particles from raw materials, proteins, and starch granules. The presence of these components limits the possibility of obtaining a stable product due to sedimentation [12]. Ultrahigh-pressure homogenization and high-pressure homogenization (HPH) are some of the novel technologies used in the production of plant-based milk substitutes. These processing technologies result in smaller and more uniform-sized particles, reduction of adverse mouthfeel, and increased stability of liquid food products [12,13]. HPH alters the arrangement of components and thus modifies the particle size, color, viscosity, and physical stability of the product [14]. Application of HPH also allows deflocculating clusters of primary oil bodies and uniform dispersion of agglomerates in plant-based beverages [14,15,16,17]. 

Since the demand for dairy alternatives is increasing in the world market, it is of primordial importance to food companies to develop new products [18]. Soybean beverage is the most popular milk substitute on the market [19,20]. However, some research studies have shown that legumes other than soybean are also suitable to produce plant-based beverages [21,22]. One of them is beans which are rich in carbohydrates, proteins, vitamins, minerals, phenolics, polyphenols, and phytosterols [15,23,24]. Legumes have gained great popularity as a dairy alternative; however, their beany flavor and the antinutritional factors (ANFs) they contain are undesirable and must therefore be eliminated in the manufacturing process [12,25]. ANFs present in food, such as phytic acid, trypsin inhibitor, lectins, and some oligosaccharides, reduce the digestibility and bioavailability of nutrients [15,26]. A variety of techniques are used to eliminate off-flavors and ANFs from legumes, including soaking, boiling, heat treatment, fermentation, and germination. During germination, proteolytic enzymes are activated, which change the protein profile of legumes. This results in an increase in the amount of protein and dietary fiber and a reduction in the content of tannins and phytic acid, as well as an improvement in the bioavailability of minerals [27,28]. Similarly, fermentation reduces off-flavors and ANFs and increases the bioavailability of bioactive components as microorganisms break down complex organic substances into simpler molecules [15,29].

The above-mentioned HPH, germination, and fermentation can aid in achieving new organoleptic and functional characteristics in the product, which can favor the production of novel dairy alternatives, including yogurt-type probiotic products [19,30,31]. Describing the properties of newly developed products using specific analytical methods is important for determining their application in the food industry. It may be advantageous to use optical methods, the application of which allows for the analysis of the stability and physicochemical characteristics of the product particles, as well as rheological methods that describe the viscosity and flow behavior of the medium. There is a lack of studies on the use of optical and rheological methods in the analysis of bean-based beverages (BBB). Therefore, our study aimed to compare the quality and storage properties of the newly developed products with colloidal-suspension structure such as yogurt-type bean-based beverages (Y-T BBB) using optical and rheological methods. The effects of germination, fermentation with three different starter cultures, and storage at 6 °C on the BBB were analyzed. Optical techniques were used to evaluate the Y-T BBB towards particle size distribution, color characteristics, and the instability phenomena during storage, while rheological tests were used to evaluate the shear viscosity, flow behavior, and viscoelastic properties. 

## 2. Materials and Methods

### 2.1. Materials and Experimental Design

The BBB used in our study were prepared from white kidney beans “Piękny Jaś Karłowy” (Lestello Sp. z o.o., Cmolas, Poland). Three industrial freeze-dried starter cultures were used: Beaugel Soja 1 (Ets Coquard, Villefranche-sur-Saône, France), which consisted of *Lactobacillus casei* (currently classified as *Lacticaseibacillus casei*), *Streptococcus thermophilus*, and *Lactobacillus delbrueckii* subsp. *bulgaricus*; YO-MIX 207 LYO 500 DCU (DuPont™ Danisco, Copenhagen, Denmark), which consisted of *S. thermophilus*, *L*. *delbrueckii* subsp. *bulgaricus*, *Lactobacillus acidophilus*, and *Bifidobacterium lactis*; and ABY-3 (Chr. Hansen, Hørsholm, Denmark), which consisted of *L. acidophilus* La-5, *Bifidobacterium animalis* subsp. *lactis* BB-12, *S*. *thermophilus*, and *L*. *delbrueckii* subsp. *bulgaricus*. 

Two variants of beverages were produced: B and BG from non- and germinated beans, respectively. The experimental design used in the study was based on three factors: beans germination, starter culture used for fermentation, and period of storage at 6 °C (Table 1). 

### 2.2. Development of Fermented Bean-Based Beverages

The BBB were obtained according to Ziarno et al. [32] with some modifications. Briefly, the germination was carried out in a sprouter at 25 °C for 72 h (water was changed every 24 h) placed in a laboratory incubator. Germinated and nongerminated beans were sterilized in the drinking water at 121 °C for 15 min to perform the starch gelatinization and inactivate microorganisms and native bean enzymes. The sterilized beans were then mixed with drinking water at a ratio of 1:9 (m/m) including the water absorbed during sterilization and blended for 7 min until a homogeneous mass was obtained. The resulting mass was filtered through a sieve with a mesh size of 0.1 mm. The beverages were then prepared by a two-step homogenization process at 50/5 MPa using a high-pressure homogenizer NS 1001 L2 PANDA, GEA Niro Soavi (GEA, Parma, Italy). HPH was used as a tool to prevent BBB destabilization according to Bernat et al. [14]. The prepared beverages were sterilized at 121 °C for 15 min.

Inoculums were prepared by dissolving the freeze-dried starter cultures in distilled water. The BBB were inoculated at 1.0% (m/m) and incubated at 45 °C for 6 h. After fermentation, the beverages were refrigerated at 6 °C and stored for 21 days. In the case of unfermented samples, 0.1% sodium azide (Chempur, Piekary Slaskie, Poland) was added to prevent the occurrence of microbiological changes during storage.

Table 2 shows the physicochemical characteristics of the developed BBB, before and after fermentation.

### 2.3. Optical Analysis

#### 2.3.1. Color Analysis

The color of the BBB was assessed using a Minolta CR-400 colorimeter (Konica Minolta Inc., Tokyo, Japan) equipped with a 2° standard observer and illuminant D65, according to the CIELab measuring system (measurement area ø = 8 mm). The color parameters determined were as follows: L (the analyzed sample was black when L = 0 or white if L = 100), a* (−a* was considered greenness and +a* redness), b* (−blue and + yellow), C* (indicating the saturation of the color), and h* (indicating the angle of the shade). The measurements were made in triplicate for each sample. The difference in the color of the samples before and after fermentation was denoted by ∆E, which expressed how far apart visually the two samples are in the color “sphere”. The reference samples for this calculation were BBB before fermentation (B0 for nongerminated beverages and BG0 for germinated beverages). The ∆E between BBB before fermentation (having $L_{1}^{*}$, $a_{1}^{*}$, $b_{1}^{*}$) and after fermentation (having $L_{2}^{*}$, $a_{2}^{*}$, $b_{2}^{*}$) was calculated according to the following equation:

(1) $$∆E=\sqrt{{\left(L_{2}^{*}-{\;L}_{1}^{*}\right)}^{2}+{\left(a_{2}^{*}-{\;a}_{1}^{*}\right)}^{2}+{\left(b_{2}^{*}-{\;b}_{1}^{*}\right)}^{2}}.$$

#### 2.3.2. Stability Analysis

The physical stability of the BBB was determined using a Turbiscan LabExpert light scattering optical analyzer (Formulation, Toulouse, France) based on the Turbiscan Stability Index (TSI) calculated using the TurbiSoftLab 2.3.1.15 program (Formulation, Toulouse, France). Briefly, 20 mL of unfermented BBB was transferred to flat-bottomed borosilicate glass cells (27.5 mm × 70 mm) and scanned immediately after inoculation. Then, the samples were fermented at 45 °C for 6 h, and kept in a laboratory refrigerator at 6 °C. The fermented BBB were stored at 6 °C and placed in the Turbiscan for scanning at preset intervals within 21 days of sample preparation. Considering backscattering profiles as a function of the height of the sample, the possible instabilities were identified and monitored at regular intervals.

#### 2.3.3. Particle Size Analysis

The particle size distribution (PSD) of the BBB was determined using a CILAS 1190 laser diffraction particle size analyzer (CILAS, Orléans, France). Measurements were performed in a recirculating cell with distilled water as a dispersant using the following operating procedures: dispersion of the sample by drops without ultrasound to achieve 15% laser obscuration, and the Fraunhofer approximation, which does not require the optical properties of the sample and preferred for opaque particles. The mean particle diameter was recorded as the volume mean diameter${\;d}_{4,3}$. PSD was characterized by ${\;d}_{V0.1}$, ${\;d}_{V0.5}$, and${\;d}_{V0.9}$ percentiles denoting points on the cumulative granulometric curve representing particle diameters at which 10%, 50%, and 90% of particles are smaller, respectively. The Span of PSD was calculated according to the following equation:

(2) $${\mathrm{Span}}_{V}=\frac{d_{V0.9}-{\;d}_{V0.1}}{d_{V0.5}}.$$

### 2.4. Rheological Analysis

Rheological measurements of the BBB were carried out using a Haake Mars 40 rheometer (Thermo Scientific, Karlsruhe, Germany). To prevent spillage, a plate with serrated platens was used (35 mm diameter, 1 mm gap) which was set at 20 °C. The results were analyzed using the HAAKE RheoWinDataManager V.4.75 software (Thermo Scientific, Karlsruhe, Germany).

Steady shear tests were performed in the controlled-rate mode at a linearly increasing shear rate of 1–100 s^−1^ for 210 s at 20 °C. Experimental flow curves (shear stress vs. shear rate) were compared using the power law model, which is the typical equation characterizing shear-thinning fluids: τ = τ_0_ + k·${\dot{\gamma }}^{n}$, where τ is the shear stress (Pa), k is the consistency index (Pa·s^n^), $\dot{\gamma }$ is the shear rate (s^−1^), τ_0_ is the yield stress (Pa), and *n* is the flow index; for a shear-thinning fluid, the values of τ_0_ and *n* are ≥0 and <1, while for a Newtonian fluid the values are 0 and 1, respectively.

Small-amplitude oscillatory shear tests were conducted at 20 °C. Two different dynamic oscillatory rheological tests were performed: strain sweep and frequency sweep. The strain amplitude with diverse strains from 0.1% to 100% was first scanned at 1 Hz in order to identify the linear viscoelastic region (LVR) for each sample. Next, the frequency-sweep test was carried out in a range of 0.1–10 Hz and a constant strain of 1%. The parameters describing the viscoelastic behavior of the samples, namely elastic moduli (G′), viscous moduli (G″), complex viscosity (|ղ*|), and loss angle tan(δ), were determined using this test. In addition, parameters “a” and “b” were determined using the power law (|ղ*| = aHz^b^) equation, where “a” is the consistency coefficient and “b” is the slope of the curve in a log–log plot of (y) against the frequency (x) and is related to the nature and behavior of the dispersion analogous to that observed for the viscous flow. The model was appropriately fit for the experimental data with a correlation coefficient (*r*^2^).

### 2.5. Statistical Analysis

The program Statistica 13.1 (StatSoft, Krakow, Poland) was used to analyze the data obtained in the experiments. Analysis of variance was used to determine the effect of germination (G), fermentation (C), and storage period (S) on the observed outcomes of the experiments. The significance of the differences was analyzed using Tukey’s test at α = 0.05.

## 3. Results and Discussion

### 3.1. Optical Analysis

Obtaining a high-quality colloidal-suspension product requires understanding the properties of its particles and its stability. It is ensured by the use of various types of optical techniques that analyze the transmissivity, reflectivity, and absorptivity of the food [33]. The optical characteristics of food have a direct impact on its properties and consumer perception [5,34]; therefore, our study used optical techniques (color, PSD, and stability analysis) to analyze the BBB.

#### 3.1.1. Color Analysis

The values of color parameters (L*, a*, b*, C*, and h*) of the BBB are shown in Table 3. Significantly (*p* < 0.05) higher L* and h* values and lower a* (i.e., in reddish), b* (i.e., in yellowness), and C* values were observed in nonfermented and fermented BG (L* ≈ 71.70–72.69, h* ≈ 81.46–81.50, a* ≈ 1.25–1.94, b* ≈ 16.78–17.03, and C* ≈ 16.88–17.04) compared to B (L* ≈ 69.81–70.49, h* ≈ 81.43–81.44, a* ≈ 2.66–2.84, b* ≈ 19.97–20.28, and C* ≈ 20.16–20.46). Although the results obtained differed significantly, their orders of magnitude remained the same; therefore, the color of both B and BG was identified as light yellow. The differences in the content of bioactive ingredients in the samples might be the reason for the different results observed in B and BG. In general, during germination, ANF levels are reduced and, consequently, the availability of bioactive ingredients is increased. It has been reported that bioactive ingredients such as phenols, carotenoids, chlorophylls, and betalains can change the color parameters of the product [35]. Moreover, BG was characterized by a significantly higher mean diameter of particles (Table 4), which might have led to higher lightness and lower color saturation in these samples. Similar to our results, other researchers have also observed that germination resulted in changes in the color parameters of products [36,37,38]. 

In both B and BG, color differences (ΔE) between fermented and unfermented samples were not higher than 1.22 (Table 3). This means that color differences cannot be easily detected by the naked human eye, and only ΔE values greater than 3.0 can be noticed [39].

#### 3.1.2. Stability and PSD Analysis

Plant-based beverages contain particulate matter that has a different density than the watery fluids they are dispersed in. This difference in density results in a gravitational pull on particulate matter. Particulate matter that is less dense than water, such as oil bodies and fat droplets, tends to reach the top surface of the beverages, whereas particulate matter with a higher density, such as plant cell fragments, starch granules, and protein aggregates, tends to sediment at the bottom [16]. The stability of plant-based beverages can be improved by reducing particle size, increasing the aqueous phase viscosity, and reducing the density contrast [16,17]. HPH has been shown to reduce the size of the particular matter [40]. 

The stability of Y-T BBB was assessed using the Turbiscan tool, which allows identifying the instability of the system in concentrated liquid dispersions and does not damage the test material [41]. The values of TSI determined after 21 days of storage at 6 °C, which stimulates the real-life conditions for Y-T BBB, are shown in Table 3. Our study showed that nonfermented and fermented B and BG, regardless of the type of the starter culture used, displayed good (category A) and satisfactory (category B) stability, respectively, in accordance with the Formulation Application Note (2013) (Table 3) [42].

**Figure 1:**
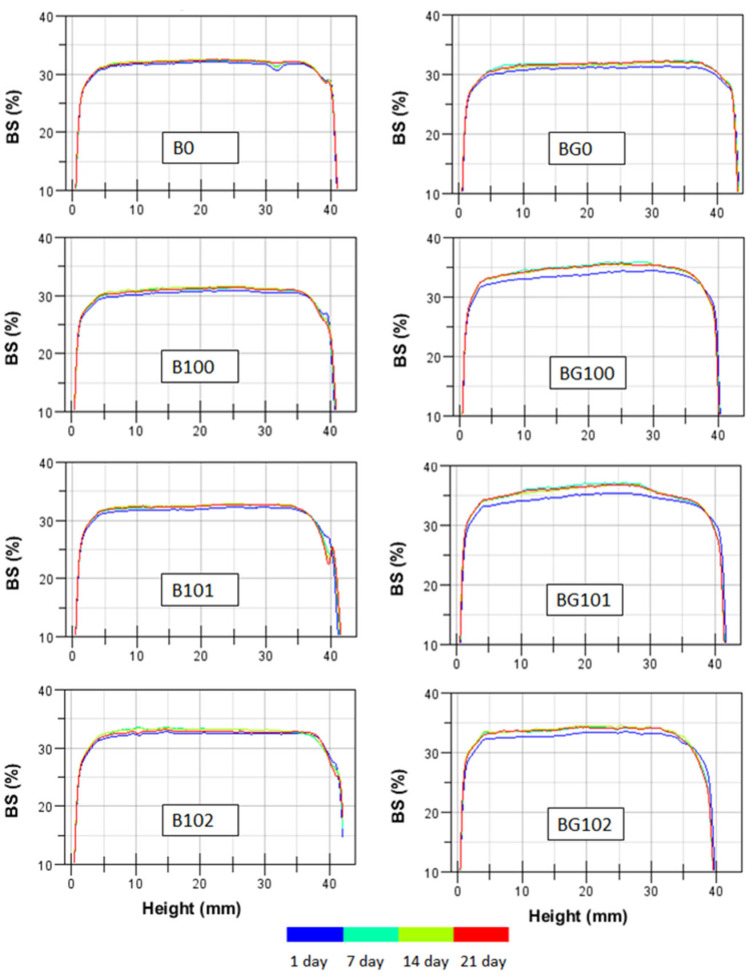
The backscatter (BS) profiles as a function of sample height within 21 days at 6 °C for BBB. Explanations: B/BG0, B/BG100, B/BG101, B/BG102—description as in Table 1.

The backscatter (BS) profiles of the BBB obtained within 21 days of storage at 6 °C are shown in Figure 1, in which the stability of the nonfermented and fermented B and BG is denoted by overlapping lines. A similar intensity in BS over the entire height of the sample was observed for all BBB. On the surface part of the sample, a decrease in BS was noticed, which may indicate the syneresis phenomenon of the sample during storage. As the temperature of the BBB decreased after fermentation, syneresis occurred due to the closer packing of starch granules [43]. An increase in BS was observed for fermented BG after 7 days of storage. During the further storage period, no changes in BS were observed, which indicates that the sample was stabilized. During the first week of storage, the pH of fermented samples could have stabilized, which resulted in changes in their BS profile. The higher availability of simple sugars due to the germination process used for BG could have increased the stabilization time of bacterial cells involved in fermentation. The lower particle size and higher viscosity of B in our study (Table 4) resulted in slightly higher stability, which is consistent with the findings of studies conducted on other systems [40,44,45,46]. 

The images of the studied nonfermented and fermented BBB stored for 21 days at 6 °C are presented in Figure S1 in the Supplementary Material. During storage, delamination of the samples was not observed and both tested types of beverages showed high storage stability.

The PSD values of B and BG before and after fermentation and after 21 days of storage are given in Table 4. HPH used in the BBB production resulted in dispersions with monomodal PSD (Figures S2 and S3 in the Supplementary Material) and with particles smaller than d_0,9_ ~ 84 µm for B and d_0,9_ ~ 170 µm for BG. Germination was the primary factor affecting the PSD of the beverages (η^2^ = 0.983). A higher Span and mean diameter were observed in the nonfermented and fermented BG (Span ≈ 2.24–2.35, d_4,3_ ≈ 76.8–84.2) compared to B (Span ≈ 1.90–2.00, d_4,3_ ≈ 38.2–47.0). Span values were used to indicate the width of the distributions and d_4,3_ as an indicator of the presence of larger particles or aggregates. The fermentation had a lesser effect (η^2^ = 0.446) on the PSD of the BBB than the germination. In BBB fermented with the ABY-3 (B/BG102) starter culture, a slight increase in d_4,3_ from 43.0 to 47.0 µm for B and from 81.5 to 82.9 µm for BG was observed.

The finest particle fraction of the BBB was probably composed mainly of proteins and oil bodies, whereas the largest particles were composed of the remains of cellular tissue and particle aggregates [14]. Nongerminated beans have a hard and dry structure, which allows more effective grinding during the processing and HPH. This results in a product with a small particle size and low particle diversity. During germination, the structure of the cell wall polysaccharides in legumes is modified, probably due to the intactness of tissue histology and disruption in the protein–carbohydrate integration. This intensifies cell wall biosynthesis and as a result, new dietary fiber is produced. As reported in a previous study, the modifications in the cell walls of the germinated seeds include changes in their physicochemical properties [47]. After germination, the beans have a more fibrous structure, which reduces the grinding efficiency and results in a product with significantly higher particle size.

Fermentation can affect the PSD of the products as the acidity of the products changes during fermentation. The decrease in pH could lead to the rearrangement of particles such as proteins and clusters on all length scales, causing particle fusion and an increase in the size of the building blocks. Formation of large protein aggregates may occur during fermentation, and this process may intensify during storage, resulting in the formation of larger particle clusters and a decrease in the number of smaller particles [48,49]. 

The values of Span differed significantly between B and BG, but those for both types of beverages showed a similar order of magnitude (~2). This indicates that the PSD of the BBB was shaped primarily during the preparation process, especially due to HPH. Other researchers have also reported that HPH increases the stability of plant-based beverages by disrupting aggregates and lipid droplets and thus decreasing their PSD. Such an effect has previously been observed for soy [50,51], hemp [40], almond, and hazelnut beverages [14] as well.

### 3.2. Rheological Analysis

Primary structure, external factors, and the time of observation are the parameters determining the rheological character of a system [52]. The BBB showed a suspension structure, which is attributable to solid particles of plant tissue of various sizes and bacterial cells present in the starter cultures. Stability studies using Turbiscan showed that the forces of interaction between the components of the colloidal suspension system of B and BG stabilized the structure of the BBB and kept them intact against weak external forces, such as the force of gravity. As deformation increases (e.g., during mixing or pumping through the conduits), the shear forces may exceed the forces of the structure-forming interaction, which leads to the reorganization of the original structure of the BBB.

In our evaluation of steady-state shear flow curves, it was assumed that each shear rate corresponds to a certain equilibrium structure and equilibrium viscosity, which allowed us to determine the relationship between the internal structure of multiphase liquid systems of the BBB and their flow properties. The viscous flow (in the range of shear rate from 1 to 100 s^−1^) of the liquid dispersions of B and BG was well described by the Ostwald–de Waele equation, which showed that all the BBB were shear-thinned fluids (*n* < 1.0) with no yield stress. The Ostwald–de Waele model parameters determined by fitting the experimental flow curves (*r*^2^ ranging from 0.994 to 0.999) are shown in Table 4. The values of *n* and k were significantly affected by germination and fermentation. The nonfermented and fermented BG showed a significantly lower consistency index k, ranging from 21.9 to 28.2 Pa·s^n^, compared to B (33.0–51.9 Pa·s^n^). After fermentation, a significant increase in k was observed for BBB fermented with ABY-3 (B/BG102) starter culture (B—from 36.9 to 49.6 Pa·s^n^, BG—from 25.7 to 28.2 Pa·s^n^). Fermentation with ABY-3 also influenced the flow index *n* in BG as a significant reduction in *n* was noticed (from 0.12 to 0.07), whereas no significant change in *n* was observed for B.

Based on the shear-thinning property of the BBB, it can be concluded that their structures were characterized by relatively low resistance to deformation and flow. Changes in the shear rate made B and BG adopt new equilibrium structures in accordance with the direction and duration of the action of shear forces. Hence, with the increase in the shear rate from 25 to 50 and 75 s^−1^, the viscosity of the nonfermented and fermented BBB systems decreased on average from 2.24 to 1.18 and 0.81 Pa·s for B and from 1.30 to 0.69 and 0.47 Pa·s for BG, respectively (Table 4). The lower viscosity and shear-thinning values of BG can be determined by PSD, which were significantly larger and had higher Span values (Table 4). In addition, studies on food suspension systems indicate that coarse particles have a flow-directional orientation [53]. 

The dispersed systems, in which a three-dimensional network of phases is observed, exhibit viscoelastic properties as a function of particle sizes [54]. Analysis of the rheological character of the BBB under dynamic shear conditions allowed us to obtain information on their nature and behavior under the influence of slight deformations.

The length of the LVR of the elastic modulus (G′) can be used as a measure of sample structure stability, since structural properties are in good correlation with elasticity. As reported in a previous study, weakly flocculated and stable dispersions have longer linear regions, whereas coagulated and strongly flocculated dispersions have relatively short linear regions [1]. The values of LVR determined in our study are presented in Table 5. The structure of the BBB was stable under low strains. The nonfermented and fermented B had a higher G′ plateau than BG, which was 191–250 Pa for B and 86–163 Pa for BG. After fermentation with starter cultures YO-MIX 207 (B101) and ABY-3 (B102), the G′ plateau was significantly increased in nonstored B (from 193 to 205–261 Pa) and significantly decreased in all fermented (BG100, BG101, and BG102) and nonstored BG (from 163 to 98–138 Pa).

**Figure 2:**
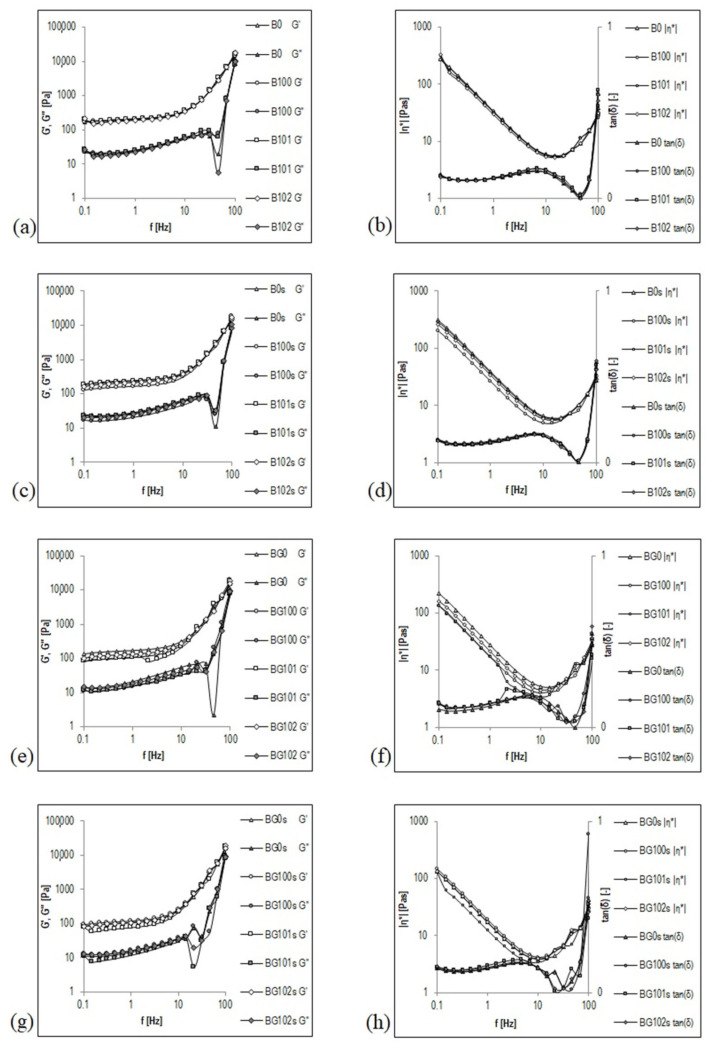
Mechanical spectra of germinated and non-germinated BBB presented graphically as functional relationships G′, G″, |η*|, and tanδ = f(Hz). Explanations: B/BG0, B/BG100, B/BG101, B/BG102—description as in Table 1.

Frequency sweep is a useful parameter to determine the viscoelastic properties of a sample as a function of timescale. It is also used to estimate several parameters, such as storage (elastic) modulus (G′) and viscous (loss) modulus (G″). G′ reflects the elastic response of a material by measuring the energy stored in the sample during the shear process, and G″ reflects the viscous response of the material by measuring the energy dissipated as heat [1]. To summarize, the frequency sweep curve gives a good rheological description of how the product will behave during storage and application. Figure 2 shows the courses of mechanical spectra G′, G″, |η*|, and tanδ for nonfermented and fermented B and BG before (Figure 2a,b,e,f) and after storage (Figure 2c,d,g,h). These figures are presented in a double logarithmic coordinate system and cover a range of three decades of oscillation frequency from f = 0.1 to 100 Hz (corresponding to ω ≈ 0.63–628 rad/s). The mechanical spectra were obtained in the range of linear viscoelasticity (1% deformation), in which the original structure of the beverages was intact.

The type of structure of the BBB was precisely and unambiguously characterized by the mechanical spectrum presented as a graph G′, G″ = f (Hz). Both nonfermented and fermented B and BG showed the character of a viscoelastic fluid with a colloidal suspension structure before and after storage. As dispersions with the structure of an entanglement network, a special feature of these viscoelastic liquids is the occurrence of a plateau in the intermediate frequency range (up to f ≈ 10 Hz), in which G′ assumes a transiently constant value, characteristic of an elastic crosslinked state (Figure 2). In the literature on rheology, this plateau is called the elastic plateau [55]. In the frequency range that involves elastic plateaus, the G″ curve appears below the G′ curve. In contrast, in relatively higher oscillation frequency ranges (third decade of the frequency from f > 10 Hz), to the right of the elastic plateau, the values of G′ and G″ modules become more dependent on the oscillation frequency, and the G′ and G″ curves follow the intersection at an oscillation frequency of ≥100 Hz. This intersection point is considered the limit of the elastic plateau, beyond which—i.e., when the curve exceeded in the range of even higher oscillation frequencies—the G″ curve will most likely run above the G′ curve [56]. This state of the multiphase system of the BBB may reflect the interactions between coarse particles present in the dispersed phase or additional energy dissipation processes resulting from the local movements of elements or fragments of the structure.

In the frequency range in which elastic plateaus appear, small values of the tangent of the loss angle tan(δ) from 0.112 to 0.149 (Figure 2b,d,f,h) express a relatively low contribution of viscous properties in the viscoelastic properties of nonfermented and fermented B and BG. The viscous features begin to appear in areas of higher oscillation frequencies, which leads to a rapid increase in the tanδ–frequency relationship. The dominant role of viscous properties in forming the viscoelastic properties of the BBB will most likely be observed at f > 10 Hz (frequency range not covered by these tests) when the values of tanδ are >1.

The complex viscosity |η*| decreases with increasing frequency in the frequency range of the elastic plateau (Figure 2b,d,f,h), which means that the BBB exhibit the properties of shear-thinning fluids. This behavior is like the one observed for these BBB in a viscous flow. The values of the coefficients a and b of the line |η*| = f(Hz) in a log–log plot in the frequency range of the elastic plateau (Table 5) correlate with the values of k (consistency coefficients) and *n* (flow index)—parameters of the Ostwald–de Waele equation describing the flow curve (Table 4). The values of both k and a are significantly higher for B (k ≈ 33–52, a ≈ 1.42–1.63) than that for BG (k ≈ 22–28, a ≈ 1.24–1.56). However, both *n* and b are significantly lower for B (*n* ≈ 0.07–0.08, b ≈ −0.087–0.090) than for BG (*n* ≈ 0.07–0.10; b ≈ −0.085–0.089). 

A significant influence of the germination on the mechanical spectra G′, G″, |η*|, and tanδ for 1 Hz was noticed in the elastic plateau (Table 5). The values of G′, G″, and |η*| were significantly higher for the nonfermented and fermented B (G′ ≈ 159–246 Pa, G″ ≈ 20–29 Pa, |η*| ≈ 25–39 Pa·s) compared to the BG (G′ ≈ 105–178 Pa, G″ ≈ 15–22 Pa, |η*| ≈ 17–29 Pa·s). This finding may be impacted by the PSD of the tested samples. The B that had a lower mean diameter d_4,3_ of the particles (Table 4) showed significantly higher G′ and G″ modules, and more complex viscosity |η*| than BG.

With respect to the rheological properties, nonfermented and fermented B and BG can be characterized as colloidal suspensions with the character of a viscoelastic fluid with the elastic plateau and also with the shear-thinning behavior in the viscous flow. Previous studies have reported similar rheological character for other liquid plant products [57,58] and dispersions such as vegetable carbon and calcium carbonate suspended in water–glycerol mixtures [59], glutenin suspensions [60], and cellulose suspensions [61]. 

## 4. Conclusions

The use of optical and rheological methods revealed that regardless of the starter cultures used in the fermentation, the BBB were characterized by a light-yellow color, high stability against dephasing during refrigerated storage, and the same rheological character. As discussed earlier, being viscoelastic fluids with a colloidal suspension structure, a special feature of both nonfermented and fermented B and BG is the occurrence of the elastic plateau in the intermediate frequency range (up to f ≈ 10 Hz), in which the BBB had a relatively low contribution of viscous properties. The viscous flow of both B and BG liquid dispersions was well described by the Ostwald–de Waele equation, which indicates that all the BBB are shear-thinned fluids with no yield stress; thus, the viscosity of the beverages decreased with an increase in shear forces.

The findings of our study demonstrate that germination is the crucial process that determines the values of selected rheological parameters (such as the parameters of the Ostwald–de-Waele model k and *n*, the viscosity ղ at shear rate of 25, 50, and 75 s^−1^, LVR, elastic (G′), and viscous (G″) moduli, complex viscosity ղ* and tan(δ) values at 1 Hz), and the TSI of the obtained beverages. This is directly related to the differences in the particle size of the particulate matter obtained by HPH of nongerminated and germinated BBB. Hence, the higher values of brightness and TSI as well as the lower values of viscosity and elastic and viscous moduli observed for BG can be explained by the PSD of the tested samples, i.e., significantly larger particles and higher Span than for B.

The applied optical and rheological methods allowed to conclude that HPH and germination mainly affected the properties of the tested plant tissue, which has a direct impact on the properties of the final products. Further research on the properties of plant-based beverages made from other plant materials is needed.

## Figures and Tables

**Table 1:** The explanation of the sample codes for B and BG beverages.

Sample Code	Beans Germination	Fermentation/Starter Culture	Storage at 6 °C
B0	-	-	1 day
B100	-	Beaugel Soja 1	1 day
B101	-	YO-MIX 207	1 day
B102	-	ABY-3	1 day
B0s	-	-	21 days
B100s	-	Beaugel Soja 1	21 days
B101s	-	YO-MIX 207	21 days
B102s	-	ABY-3	21 days
BG0	+	-	1 day
BG100	+	Beaugel Soja 1	1 day
BG101	+	YO-MIX 207	1 day
BG102	+	ABY-3	1 day
BG0s	+	-	21 days
BG100s	+	Beaugel Soja 1	21 days
BG101s	+	YO-MIX 207	21 days
BG102s	+	ABY-3	21 days

**Table 2:** Physicochemical characteristics of for B and BG beverages.

Sample Code ^1^	pH	Dry Mater Content (g/100 g)	Water Activity (–)
B0	5.86	9.41	0.998
B100	5.86	8.74	0.997
B101	4.46	8.61	0.992
B102	4.14	8.25	0.994
BG0	6.46	7.33	0.996
BG100	4.41	6.66	0.995
BG101	4.27	6.89	0.995
BG102	4.13	7.13	0.996

^1^ Description as in Table 1.

**Table 3:** Variations of color parameters L*, a*, b*, C*, h* and color difference (∆E) between BBB before fermentation and after fermentation, and the results of Turbiscan Stability Index (TSI) after 21 days of storage at 6 °C.

Sample Code ^1^	Color Parameters						Turbiscan Stability	
	L*	a*	b*	C*	h*	∆E	TSI	Category ^2^
B0	70.49 ± 0.01 ^c^	2.66 ± 0.02 ^c^	20.28 ± 0.03 ^e^	20.46 ± 0.02 ^e^	81.44 ± 0.00 ^b^	-	0.65 ± 0.10 ^a^	A
B100	69.81 ± 0.01 ^a^	2.79 ± 0.02 ^cd^	19.97 ± 0.02 ^c^	20.16 ± 0.03 ^c^	81.43 ± 0.00 ^ab^	0.77 ± 0.02 ^b^	0.75 ± 0.10 ^a^	A
B101	70.18 ± 0.01 ^b^	2.84 ± 0.00 ^d^	20.11 ± 0.01 ^d^	20.31 ± 0.01 ^d^	81.43 ± 0.00 ^a^	0.41 ± 0.03 ^a^	0.85 ± 0.20 ^a^	A
B102	70.22 ± 0.01 ^b^	2.84 ± 0.02 ^d^	20.18 ± 0.01 ^de^	20.45 ± 0.01 ^e^	81.44 ± 0.00 ^b^	0.35 ± 0.02 ^a^	0.90 ± 0.10 ^a^	A
BG0	71.70 ± 0.07 ^d^	1.25 ± 0.17 ^a^	17.03 ± 0.11 ^b^	17.04 ± 0.12 ^b^	81.50 ± 0.01 ^d^	-	1.40 ± 0.30 ^ab^	B
BG100	72.50 ± 0.04 ^e^	1.93 ± 0.01 ^b^	16.89 ± 0.03 ^ab^	16.99 ± 0.02 ^ab^	81.46 ± 0.00 ^c^	1.07 ± 0.16 ^bc^	1.45 ± 0.10 ^ab^	B
BG101	72.69 ± 0.02 ^f^	1.91 ± 0.01 ^b^	16.78 ± 0.01 ^a^	16.88 ± 0.01 ^a^	81.46 ± 0.00 ^c^	1.22 ± 0.14 ^c^	2.00 ± 0.20 ^b^	B
BG102	72.65 ± 0.04 ^f^	1.94 ± 0.02 ^b^	16.89 ± 0.06 ^ab^	16.97 ± 0.07 ^ab^	81.46 ± 0.00 ^c^	1.18 ± 0.17 ^c^	1.30 ± 0.10 ^ab^	B
Statistics ANOVA, η2 [-]								
G	0.933	0.945	0.998	0.999	0.777	0.813	0.701	-
C	ns	0.688	0.661	0.568	0.604	ns	ns	-

^a, b, c, d, e, f^—mean values in columns denoted by different letters differ significantly (*p* ≤ 0.05). ^1^ Description as in Table 1. ^2^ Category of Turbiscan Stability and TSI limits: A+—excellent stability (TSI < 0.5), A—good stability (TSI 0.5–1.0), B—satisfactory stability (TSI 1.0–3.0), C—poor stability (TSI 3.0–10.0), D—unsatisfactory stability (TSI > 10.0). Explanations: ns—non-significant; G—germination; C—starter culture; S—storage period; η^2^—coefficient indicating the extent of the effect of factors G and C.

**Table 4:** The results of the volume PSD (d_4,3_, d_0,9_, Span) and summary of parameter values related to flow (the parameters of the Ostwald-de-Waele model k and *ո*, and the viscosity ղ at shear rate of 25, 50 and 75 s^−1^) obtained for BBB before fermentation, after fermentation, and after 21 days of storage.

Sample Code ^1^	$d_{4,3}\;(µm)$	$d_{0,9}\;(µm)$	Span (-)	k (Pa·s^n^)	*n* (–)	ղ at 25 s^−1^ (Pa·s)	ղ at 50 s^−1^ (Pa·s)	ղ at 75 s^−1^ (Pa·s)
B0	43.0 ± 0.1 ^ab^	84.3 ± 0.5 ^ab^	2.00 ± 0.04 ^a^	36.9 ± 1.4 ^d^	0.08 ± 0.01 ^abc^	1.94 ± 0.01 ^d^	1.03 ± 0.02 ^e^	0.71 ± 0.01 ^e^
B100	38.2 ± 0.2 ^a^	74.1 ± 0.5 ^a^	1.94 ± 0.01 ^a^	33.0 ± 2.1 ^cd^	0.08 ± 0.01 ^ab^	1.70 ± 0.06 ^c^	0.89 ± 0.03 ^d^	0.62 ± 0.02 ^d^
B101	44.9 ± 0.3 ^ab^	87.4 ± 0.5 ^b^	1.96 ± 0.01 ^a^	50.0 ± 2.8 ^ef^	0.07 ± 0.01 ^a^	2.50 ± 0.09 ^gh^	1.31 ± 0.04 ^ghi^	0.90 ± 0.03 ^gh^
B102	47.0 ± 0.1 ^b^	92.0 ± 0.4 ^b^	1.98 ± 0.01 ^a^	49.6 ± 2.1 ^ef^	0.08 ± 0.01 ^ab^	2.56 ± 0.04 ^gh^	1.35 ± 0.02 ^hi^	0.93 ± 0.01 ^hi^
B0s	46.6 ± 0.3 ^b^	90.1 ± 0.1 ^b^	1.92 ± 0.03 ^a^	45.1 ± 1.2 ^e^	0.08 ± 0.00 ^ab^	2.34 ± 0.05 ^f^	1.24 ± 0.03 ^g^	0.85 ± 0.02 ^g^
B100s	41.6 ± 0.1 ^ab^	81.2 ± 0.1 ^ab^	1.97 ± 0.01 ^a^	36.9 ± 0.9 ^d^	0.07 ± 0.00 ^a^	1.86 ± 0.03 ^d^	0.98 ± 0.01 ^e^	0.67 ± 0.01 ^de^
B101s	45.0 ± 0.1 ^ab^	86.4 ± 0.1 ^ab^	1.90 ± 0.02 ^a^	47.1 ± 1.0 ^ef^	0.08 ± 0.01 ^ab^	2.43 ± 0.04 ^fg^	1.28 ± 0.02 ^gh^	0.88 ± 0.02 ^gh^
B102s	46.2 ± 0.2 ^b^	89.2 ± 0.4 ^b^	1.91 ± 0.01 ^a^	51.9 ± 0.3 ^ef^	0.07 ± 0.00 ^ab^	2.63 ± 0.02 ^h^	1.38 ± 0.01 ^i^	0.95 ± 0.01 ^i^
BG0	81.5 ± 1.6 ^c^	169.8 ± 5.5 ^d^	2.35 ± 0.06 ^b^	25.7 ± 2.3 ^ab^	0.12 ± 0.01 ^c^	1.48 ± 0.10 ^e^	1.12 ± 0.05 ^f^	0.56 ± 0.04 ^f^
BG100	76.8 ± 0.2 ^c^	156.8 ± 0.6 ^c^	2.25 ± 0.01 ^b^	22.4 ± 0.8 ^a^	0.10 ± 0.00 ^bc^	1.25 ± 0.05 ^a^	0.67 ± 0.02 ^abc^	0.53 ± 0.02 ^abc^
BG101	78.3 ± 1.2 ^c^	160.8 ± 3.1 ^cd^	2.27 ± 0.01 ^b^	25.9 ± 1.0 ^ab^	0.08 ± 0.01 ^ab^	1.33 ± 0.05 ^ab^	0.70 ± 0.03 ^bc^	0.48 ± 0.02 ^bc^
BG102	82.9 ± 0.1 ^cd^	171.3 ± 1.7 ^d^	2.29 ± 0.04 ^b^	28.2 ± 1.9 ^bc^	0.07 ± 0.01 ^a^	1.42 ± 0.06 ^b^	0.75 ± 0.03 ^c^	0.51 ± 0.02 ^c^
BG0s	79.3 ± 0.2 ^c^	161.9 ± 0.1 ^cd^	2.25 ± 0.01 ^b^	21.9 ± 0.3 ^a^	0.10 ± 0.00 ^bc^	1.23 ± 0.01 ^a^	0.66 ± 0.01 ^ab^	0.46 ± 0.00 ^ab^
BG100s	80.1 ± 0.1 ^c^	163.5 ± 0.4 ^cd^	2.24 ± 0.01 ^b^	24.3 ± 0.6 ^ab^	0.08 ± 0.00 ^ab^	1.27 ± 0.02 ^ab^	0.67 ± 0.01 ^abc^	0.46 ± 0.00 ^abc^
BG101s	84.2 ± 0.1 ^d^	173.9 ± 0.1 ^cd^	2.28 ± 0.01 ^b^	23.2 ± 0.8 ^a^	0.09 ± 0.01 ^abc^	1.25 ± 0.02 ^a^	0.66 ± 0.01 ^ab^	0.46 ± 0.01 ^ab^
BG102s	82.9 ± 0.6 ^cd^	169.1 ± 1.4 ^d^	2.23 ± 0.01 ^b^	24.2 ± 0.5 ^ab^	0.07 ± 0.00 ^a^	1.20 ± 0.05 ^a^	0.63 ± 0.00 ^a^	0.43 ± 0.00 ^a^
Statistics ANOVA, η^2^ (-)								
G	0.983	0.989	0.960	0.876	0.217	0.739	0.733	0.729
C	0.446	0.469	ns	0.500	0.347	0.307	0.302	0.299
S	0.177	ns	0.279	ns	ns	ns	ns	ns

^a, b, c, d, e, f, g, h, i^—mean values in columns denoted by different letters differ significantly (*p* ≤ 0.05). ^1^ Description as in Table 1. Explanations: ns—non-significant; G—germination; C—starter culture; S—storage period; η^2^—coefficient indicating the extent of the effect of factors G, C, and S.

**Table 5:** Rheological parameters under oscillatory testing at 20 °C (including: LVR values obtained in the amplitude sweep test at 1Hz; elastic (G′) and viscous (G″) moduli, complex viscosity ղ*, and tan(δ) values at 1 Hz obtained at the frequency sweep test at strain 1%; and parameters obtained by a power law (|ղ*| = aHz^b^) equation of the curve in a log–log plot) for the BBB before and after 21 days of storage.

Sample Code ^1^	LVR		Frequency Sweep. Values at 1 Hz				log(ղ*) = a + blog(Hz) at f < 10 Hz		
	G′ Plateau (Pa)	γ (%)	G′ (Pa)	G″ (Pa)	|ղ*| (Pa·s)	tan(δ) (–)	a	b	r^2^
B0	193 ± 25 ^cde^	2.3 ± 0.4 ^ab^	203 ± 15 ^abcd^	24 ± 1 ^ab^	33 ± 2 ^abcd^	0.120 ± 0.004 ^a^	1.52 ± 0.03 ^bc^	−0.888 ± 0.004 ^abc^	1.000 ± 0.000 ^a^
B100	183 ± 11 ^cde^	2.1 ± 0.2 ^ab^	159 ± 43 ^abcd^	20 ± 4 ^ab^	26 ± 6 ^abcd^	0.126 ± 0.007 ^abc^	1.42 ± 0.11 ^abc^	−0.889 ± 0.009 ^abc^	0.998 ± 0.003 ^a^
B101	205 ± 10 ^def^	2.8 ± 0.3 ^ab^	198 ± 16 ^abcd^	24 ± 2 ^ab^	32 ± 3 ^abcd^	0.120 ± 0.001 ^a^	1.52 ± 0.03 ^bc^	−0.897 ± 0.006 ^bc^	0.998 ± 0.002 ^a^
B102	261 ± 20 ^g^	2.5 ± 0.3 ^ab^	229 ± 37 ^bcd^	26 ± 3 ^ab^	37 ± 6 ^bcd^	0.113 ± 0.004 ^a^	1.58 ± 0.07 ^d^	−0.898 ± 0.005 ^bc^	0.999 ± 0.002 ^a^
B0s	191 ± 16 ^cde^	2.0 ± 0.1 ^a^	193 ± 45 ^abcd^	24 ± 4 ^ab^	31 ± 6 ^abcd^	0.124 ± 0.006 ^ab^	1.50 ± 0.09 ^bc^	−0.902 ± 0.009 ^c^	0.996 ± 0.004 ^a^
B100s	176 ± 5 ^cd^	2.0 ± 0.1 ^a^	159 ± 7 ^abcd^	20 ± 1 ^ab^	25 ± 1 ^abcd^	0.125 ± 0.001 ^ab^	1.42 ± 0.02 ^abc^	−0.873 ± 0.004 ^abc^	0.999 ± 0.000 ^a^
B101s	231 ± 14 ^efg^	2.3 ± 0.1 ^ab^	260 ± 32 ^d^	29 ± 3 ^b^	42 ± 6 ^d^	0.112 ± 0.002 ^a^	1.63 ± 0.05 ^d^	−0.895 ± 0.007 ^bc^	1.000 ± 0.000 ^a^
B102s	250 ± 27 ^fg^	2.3 ± 0.2 ^ab^	246 ± 51 ^cd^	28 ± 5 ^b^	39 ± 7 ^cd^	0.116 ± 0.003 ^a^	1.60 ± 0.08 ^d^	−0.889 ± 0.001 ^abc^	1.000 ± 0.000 ^a^
BG0	163 ± 17 ^bcd^	2.9 ± 0.2 ^ab^	178 ± 25 ^abcd^	22 ± 5 ^ab^	29 ± 7 ^abcd^	0.126 ± 0.005 ^abc^	1.56 ± 0.09 ^bc^	−0.889 ± 0.001 ^bc^	1.000 ± 0.000 ^a^
BG100	98 ± 22 ^a^	3.0 ± 0.1 ^b^	147 ± 37 ^abc^	19 ± 4 ^ab^	24 ± 8 ^abc^	0.137 ± 0.013 ^bcd^	1.24 ± 0.06 ^a^	−0.850 ± 0.005 ^a^	0.998 ± 0.001 ^a^
BG101	112 ± 18 ^ab^	2.5 ± 0.1 ^ab^	113 ± 23 ^a^	16 ± 3 ^ab^	18 ± 4 ^a^	0.144 ± 0.004 ^d^	1.27 ± 0.09 ^a^	−0.875 ± 0.005 ^abc^	0.997 ± 0.002 ^a^
BG102	138 ± 10 ^abc^	2.7 ± 0.5 ^ab^	135 ± 27 ^ab^	19 ± 3 ^ab^	22 ± 4 ^ab^	0.143 ± 0.003 ^cd^	1.34 ± 0.09 ^ab^	−0.876 ± 0.009 ^abc^	0.999 ± 0.000 ^a^
BG0s	86 ± 3 ^a^	2.8 ± 0.5 ^ab^	108 ± 20 ^a^	16 ± 3 ^a^	17 ± 3 ^a^	0.145 ± 0.005 ^d^	1.26 ± 0.07 ^a^	−0.851 ± 0.008 ^ab^	0.998 ± 0.001 ^a^
BG100s	109 ± 7 ^ab^	2.7 ± 0.1 ^ab^	111 ± 7 ^a^	16 ± 1 ^a^	18 ± 1 ^a^	0.142 ± 0.002 ^cd^	1.28 ± 0.03 ^a^	−0.853 ± 0.004 ^abc^	0.998 ± 0.000 ^a^
BG101s	110 ± 10 ^ab^	2.5 ± 0.5 ^ab^	105 ± 24 ^a^	15 ± 4 ^a^	17 ± 5 ^a^	0.149 ± 0.011 ^d^	1.25 ± 0.09 ^a^	−0.857 ± 0.008 ^abc^	0.996 ± 0.003 ^a^
BG102s	108 ± 10 ^a^	2.8 ± 0.2 ^ab^	108 ± 8 ^a^	16 ± 1 ^a^	17 ± 1 ^a^	0.145 ± 0.005 ^d^	1.27 ± 0.01 ^a^	−0.847 ± 0.003 ^a^	0.998 ± 0.000 ^a^
Statistics ANOVA, η2 [-]									
G	0.792	0.350	0.509	0.425	0.508	0.715	0.714	0.456	ns
C	0.328	ns	ns	ns	ns	ns	ns	ns	ns
S	ns	ns	ns	ns	ns	ns	ns	ns	ns

^a, b, c, d, e, f, g^—mean values in columns denoted by different letters differ significantly (*p* ≤ 0.05). ^1^ Description as in Table 1. Explanations: ns—non-significant; G—germination; C—starter culture; S—storage period; η^2^—coefficient indicating the extent of the effect of factors G, C, and S.

## Data Availability

Not applicable.

## Supplementary Materials

The following supporting information can be downloaded at: https://www.mdpi.com/article/10.3390/s22218348/s1, Figure S1: The tested bean-based beverages before and after 21 days of storage period at 6 °C; Figure S2: The particle size distribution of the nongerminated bean-based beverages; Figure S3: The particle size distribution of the germinated bean-based beverages.
