# Supplementary material for: Application of Optical and Rheological Techniques in Quality and Storage Assessment of the Newly Developed Colloidal-Suspension Products: Yogurt-Type Bean-Based Beverages
Source: Sensors (Basel). 2022 Oct 31;22(21):8348. doi: 10.3390/s22218348 (PMC9656516; doi:10.3390/s22218348)
Supplement: Supplementary file 1 [file sensors-22-08348-s001.zip › sensors-1994037-supplementary.pdf]

SUPPLEMENTARY MATERIAL

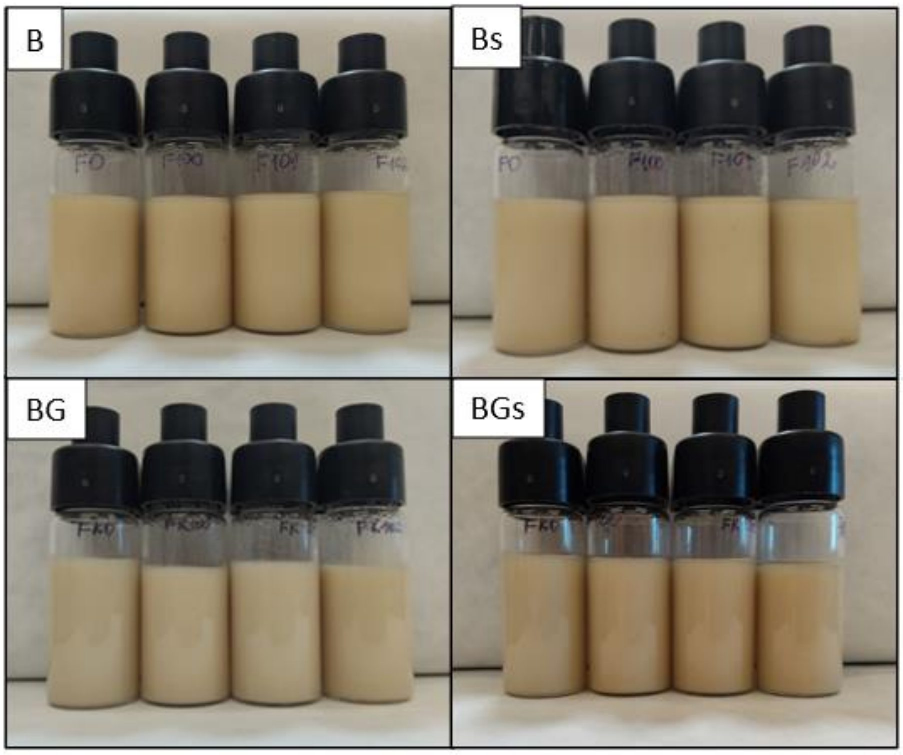

Figure S1. The tested BBB before and after 21-days of storage.

Explanations: B – non-germinated BBB before storage, Bs - non-germinated BBB after 21 days of storage, BG - germinated BBB before storage, BGs - germinated BBB after 21 days of storage**.**

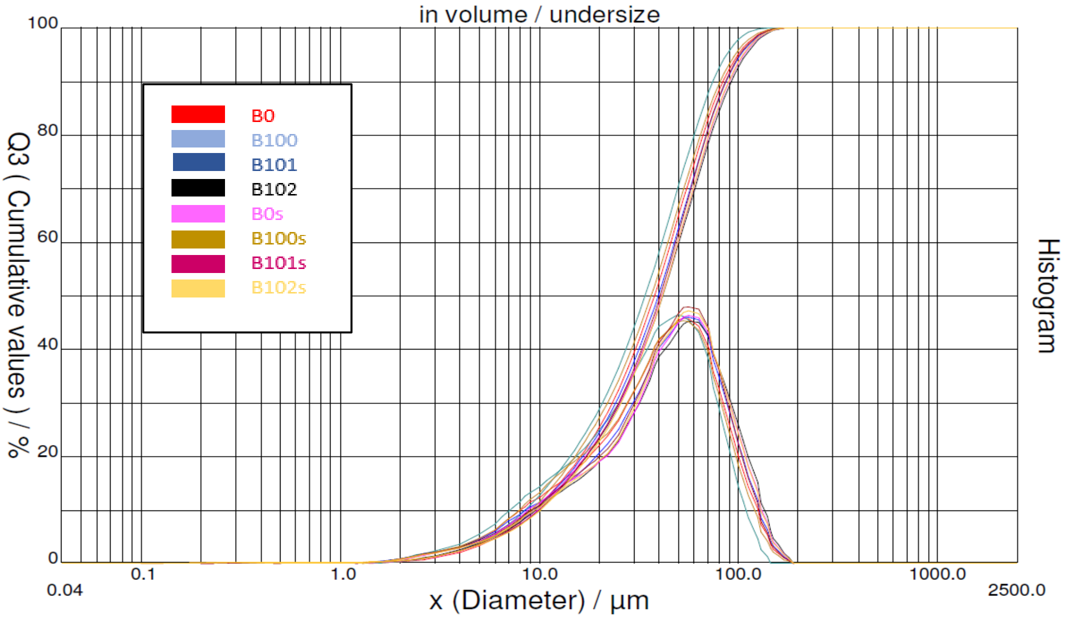

Figure S2. The particle size distribution of the non-germinated BBB.
Explanations: B/BG0, B/BG100, B/BG101, B/BG102 - description as in Table 1.

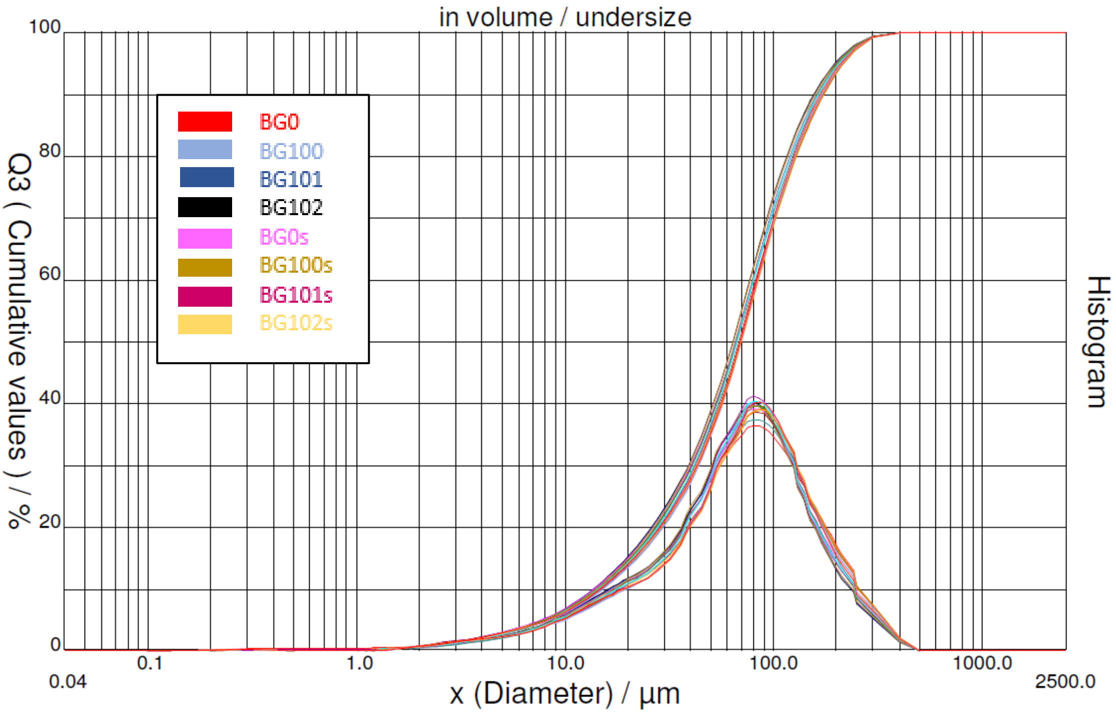

Figure S3. The particle size distribution of the germinated BBB.
Explanations: B/BG0, B/BG100, B/BG101, B/BG102 - description as in Table 1.
